# Impact of ventricular arrhythmia management on suboptimal biventricular pacing in cardiac resynchronization therapy

**DOI:** 10.1007/s10840-022-01259-0

**Published:** 2022-06-14

**Authors:** Jan-Hendrik van den Bruck, Melissa Middeldorp, Arian Sultan, Cornelia Scheurlen, Katharina Seuthe, Jonas Wörmann, Karlo Filipovic, Kadhim Kadhim, Prashanthan Sanders, Daniel Steven, Jakob Lüker

**Affiliations:** 1 Department of Electrophysiology, University of Cologne, Faculty of Medicine and University Hospital Cologne, Kerpener Strasse 62, 50937 Cologne, Germany; 2 Centre for Heart Rhythm Disorders, University of Adelaide and Royal Adelaide Hospital, Adelaide, Australia

**Keywords:** Catheter ablation of ventricular ectopy, Optimization of Cardiac resynchronization therapy, Catheter ablation for Optimization of biventricular pacing

## Abstract

**Background:**

Reduced biventricular pacing (BiVP) is a common phenomenon in cardiac resynchronization therapy (CRT) with impact on CRT-response and patients’ prognosis. Data on treatment strategies for patients with ventricular arrhythmia and BiVP reduction is sparse. We sought to assess the effects of ventricular arrhythmia treatment on BiVP.

**Methods:**

In this retrospective analysis, the data of CRT patients with a reduced BiVP ≤ 97% due to ventricular arrhythmia were analyzed. Catheter ablation or intensified medical therapy was performed to optimize BiVP.

**Results:**

We included 64 consecutive patients (73 ± 10 years, 89% male, LVEF 30 ± 7%). Of those, 22/64 patients (34%) underwent ablation of premature ventricular contractions (PVC) and 15/64 patients (23%) underwent ventricular tachycardia (VT) ablation while 27/64 patients (42%) received intensified medical treatment. Baseline BiVP was 88.1% ± 10.9%. An overall increase in BiVP percentage points of 8.8% (range − 5 to + 47.6%) at 6-month follow-up was achieved. No changes in left ventricular function were observed but improvement in BiVP led to an improvement in NYHA class in 24/64 patients (38%). PVC ablation led to a significantly better improvement in BiVP [9.9% (range 4 to 22%) vs. 3.2% (range − 5 to + 10.7%); *p* =  < 0.001] and NYHA class (12/22 patients vs. 4/27 patients; *p* = 0.003) than intensified medical therapy. All patients with VT and reduced BiVP underwent VT ablation with an increase of BiVP of 16.3 ± 13.4%.

**Conclusion:**

In this evaluation of ventricular arrhythmia treatment aiming for CRT optimization, both medical therapy and catheter ablation were shown to be effective. Compared to medical therapy, a higher increase in BiVP was observed after PVC ablation, and more patients improved in NYHA class.

**Clinical Trial Registration:**

The study was registered at clinical trials.org in August 2019: NCT04065893.

## Introduction

Cardiac resynchronization therapy (CRT) is well established in patients with symptomatic heart failure, reduced left ventricular ejection fraction (LVEF), and wide QRS complex [1]. CRT leads to an improvement of functional status, echocardiographic parameters, and most importantly to a reduction of mortality [2][2]. These beneficial effects of CRT are rooted in the elimination of dyssynchrony, and improvement of cardiac function. They fundamentally depend on the efficacy and percentage of biventricular pacing (BiVP) [4, 5].

Impairment of BiVP is common in CRT follow-up care. Besides device malfunction and lead failure, atrial arrhythmias, device programming, ventricular tachycardia, and premature ventricular contractions (PVC) are the most common causes of a reduction of BiVP [6]. Due to the strong relationship between outcome and BiVP, surveillance and optimization of BiVP, aiming for a percentage $\ge$ 98%, are a key challenge in CRT follow-up care [4].

While reduction of BiVP due to atrial arrhythmias often leads to a sudden and drastic dip, ventricular arrhythmias commonly lead to a prolonged and minor reduction of BiVP [6].

Nevertheless, ventricular ectopy is associated with a higher mortality and morbidity in CRT patients, potentially as a result of reduced BiVP [7]. Consequently, a reduced BiVP due to ventricular arrhythmia requires either medical or interventional treatment. Despite its evident impact on patient outcome, there is no robust data available regarding the optimal treatment strategy of ventricular arrhythmia–induced BiVP reduction.

Catheter ablation of ventricular ectopy from the right ventricular outflow tract was shown to be more effective than medical therapy. It improved mortality and morbidity in patients with left ventricular (LV) dysfunction, and CRT response in nonresponders [8–10]. Ventricular tachycardia (VT) ablation is an established therapy in defibrillator patients suffering defibrillator therapy despite antiarrhythmic medication [11, 12].

There is still a relevant number of patients, irrespective of their initial response status, experiencing a loss of BiVP over time due to complex ventricular ectopy or ventricular tachycardia without defibrillator therapy [13].

To the best of our knowledge, this retrospective and non-randomized two-center registry provides the first evaluation of different treatment options of ventricular arrhythmias aiming for CRT optimization and improvement of biventricular pacing.

## Methods

### Inclusion criteria and study population

All CRT patients aged ≥ 18 years with a BiVP $\le$ 97% presenting to the outpatient clinic of the University Hospital of Cologne and the Centre for Heart Rhythm Disorders, University of Adelaide, between 01/2019 and 02/2021 were analyzed. All patients with a reduced BiVP due to ventricular arrhythmia, irrespective of their initial response status, were included in this retrospective analysis. Patients with a BiVP reduction related to other reasons were excluded. In patients with a history of atrial fibrillation, a thorough analysis was performed to exclude atrial fibrillation as the underlying cause of BiVP reduction. Only patients with no episodes of atrial fibrillation in the device Holter or patients, which had previously undergone AV-node ablation, were included in this registry. When ventricular ectopy was the suspected cause of BiVP reduction, a conservative pharmacological approach or catheter ablation of ventricular ectopy was performed to optimize biventricular pacing. The decision to perform either PVC ablation or intensify medical therapy was made at the treating physician’s discretion for the individual patient. In patients with repeated episodes of non-sustained ventricular tachycardia, recorded in the Device Holter, or episodes of hemodynamically stable slow VT below the detection threshold and consequently reduced BiVP, a VT ablation was performed. Respective patients were analyzed separately. Patients with VT and ICD therapy were excluded. The study was approved by the Institutional Review Board and registered at clinical trials.org (NCT04065893).

### Catheter ablation procedures

All ablation procedures were guided by a 3D navigation system using the CARTO® (Biosense Webster, Diamond Bar, CA, USA) or EnSite Precision™ (Abbott, St. Paul, MN, USA) mapping system. Procedures were performed via transfemoral access and under intravenous sedation. In all LV procedures, a retrograde, transseptal, or combined access was conducted. Heparin was administered targeting an activated clotting time > 300 s [10].

A multipolar mapping catheter (Pentaray®, Biosense Webster or Advisor™ HD Grid, Abbott) was employed at the operator’s discretion. Radiofrequency ablation was performed with an irrigated catheter (Navistar Thermocool®, Biosense Webster or Flexability™ Sensor Enabled, Abbott). Contact force–measuring catheters (Thermocool Smart Touch® Surround Flow, Biosense Webster or TactiCath™ Sensor Enabled, Abbott) were used at the operator’s discretion.

Anatomic and activation mapping was conducted simultaneously, when possible. Voltage thresholds were defined as follows: $<$ 0.5 mV as scar, 0.5 to 1.5 mV as border zone. A combination of pace mapping with automated matching (CARTO® PASO®, Biosense Webster, EnSite Precision™ Automap™) and activation mapping was the aspired mapping strategy for all cases [10]. In all VT ablation procedures, induction via programmed right ventricular stimulation was conducted and a substrate-based approach was performed if the tachycardia was hemodynamically not tolerated.

In the absence of spontaneous ectopy, intravenous isoproterenol was administered, sedation was reduced, or a hand-grip maneuver was performed. If ectopy remained infrequent, a pace-mapping–based approach assisted by automated template matching was performed [14]. Complete elimination of all PVC/VT morphologies was targeted whenever possible. If more than one PVC/VT morphology was present, the clinically predominant PVC/VT was targeted first. After the last radiofrequency application, patients were monitored for a minimum of 30 min to ensure cessation of the ventricular ectopy. In VT ablations, repeated reinduction was performed to evaluate non-inducibility of all VT.

### Intensified medical therapy

In all CRT patients with a BiVP percentage $\le$ 97% not receiving invasive treatment, a preexisting betablocker therapy was intensified aiming for the recommended maximum daily dosage. Moreover, in a small subgroup of patients an oral amiodarone treatment was initialized with 600 mg per day for 14 days, followed by 400 mg for additional 14 days. Afterwards, a maintenance dose of 200 mg per day was administered.

### Follow-up

During consecutive routine follow-up visits every 3 months, all patients underwent device interrogation, 12-lead ECG and clinical evaluation at the respective center’s outpatient clinic. After 6 months of follow-up BiVP, functional status and LV-EF were reassessed, and the impact of measures taken to optimize BiVP was evaluated.

### Statistical analysis

Data analysis was performed using SPSS statistical software (version 26). Data are shown in absolute values, percentages, medians with range, and means with standard deviation. Variables were tested for normal distribution by the Shapiro–Wilk test. For the comparison of continuous variables, the Student’s *t*-test or Mann–Whitney *U*-test were used. Categorical variables were compared using contingency tables and application of the chi-square test or Fisher’s exact test. *p*-values < 0.05 were considered statistically significant.

## Results

### Study population

**Fig. 1:**
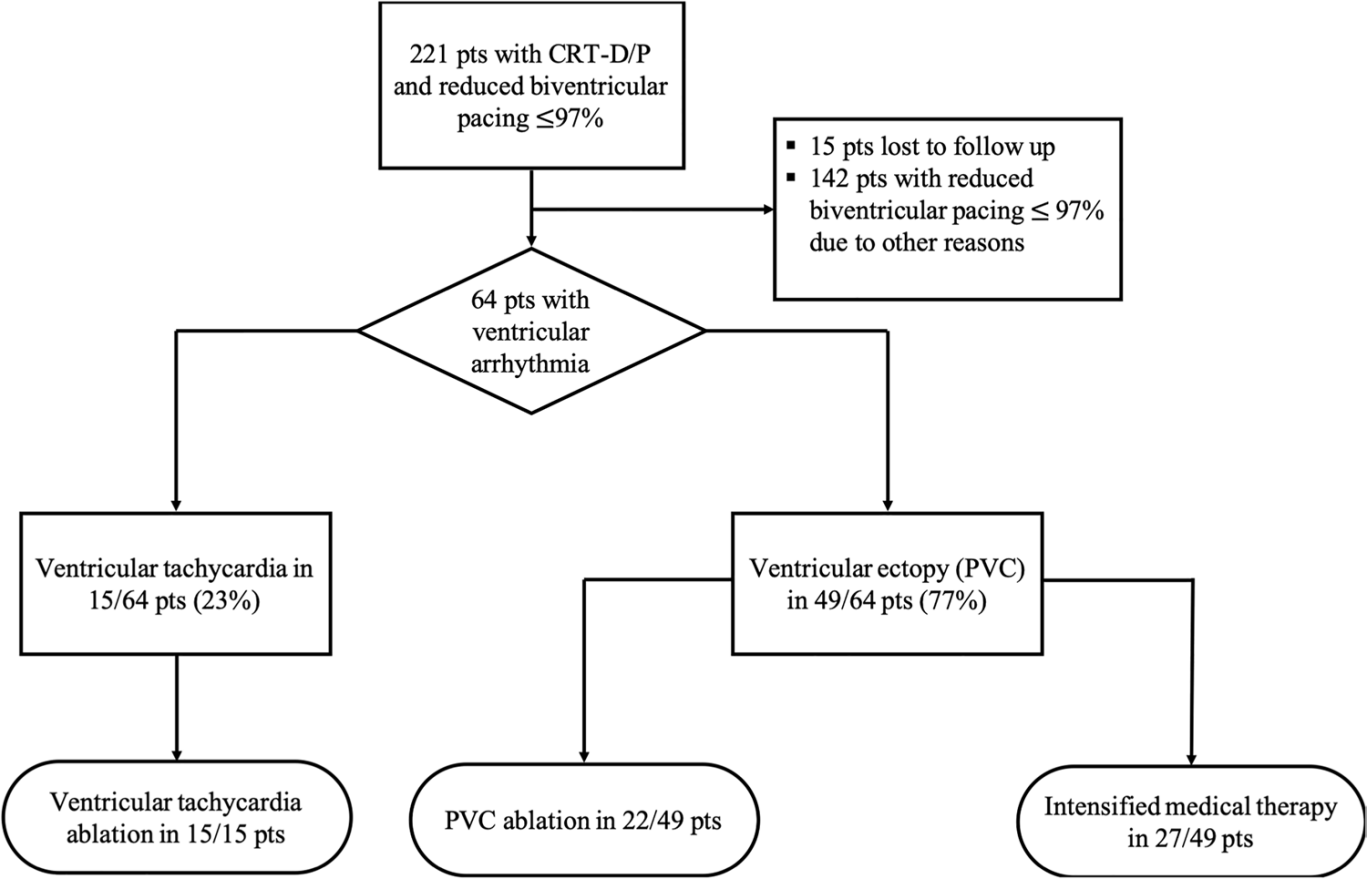
Study design. A total of 64 CRT patients (pts) presented a reduced biventricular pacing (BiVP) percentage $\le$ 97% due to ventricular arrhythmia. Of those in 49/64 pts, BiVP was impaired by ventricular ectopy. Consequently, 22/49 pts underwent ablation of ventricular ectopy and 27/49 received intensified medical therapy aiming for optimization of BiVP. All pts with BiVP reduction due to ventricular tachycardia (VT) underwent VT ablation

A total of 221 patients with a CRT device and a BiVP percentage $\le$ 97% were analyzed. Of those, 15 (7%) were lost to follow-up and in 142 (64%) the reduction of BiVP was due to reasons other than ventricular arrhythmia. Consequently, 64 patients (age 73.1 ± 10.3 years, 89% male) were included in this analysis (Fig. 1). Of those, 22 patients underwent PVC ablation and 27 patients received intensified medical therapy aiming for PVC suppression. Baseline characteristics and medication are provided in Table 1 and did not differ significantly between both PVC treatment groups. Ablation of VT was undertaken in 15 patients (age 72.3 ± 9.7 years, 100% male). Of those, 6/15 patients (40%) had repeated non-sustained VTs and 9/15 (60%) VTs below detection threshold (Table 1). In 8/15 patients, these respective VT episodes occurred under a preexisting amiodarone therapy. No additional amiodarone therapy was initiated after the ablation procedure.

**Table 1:** Baseline characteristics and cardiovascular medication. Means and standard deviation or absolute numbers and percentage are shown

Parameter	All	PVC ablation group	VT ablation group	Intensified medical therapy group	*p*-value
Sex (male)	57/64 (89%)	18/22 (82%)	15/15	24/27 (89%)	0.7
Age (years)	73.1 ± 10.3	70.7 ± 10.7	73.2 ± 9.8	75.5 ± 10.1	0.1
BMI (kg/m^2^)	27.3 ± 5.3	26.7 ± 4.2	27.8 ± 3.2	27.4 ± 6.1	0.7
Coronary heart disease	42/64 (65%)	13/22 (59%)	11/15 (73%)	18/27 (67%)	0.8
Diabetes	15/64 (23%)	5/22 (23%)	5/15 (33%)	5/27 (19%)	0.7
Atrial fibrillation	26/64 (41%)	8/22 (36%)	10/15 (67%)	8/27 (30%)	0.7
Renal failure	42/64 (66%)	13/22 (59%)	9/15 (60%)	20/27 (74%)	0.4
Reason for CRT					
Ischemic cardiomyopathy	39/64 (61%)	11/22 (50%)	11/15 (73%)	17/27 (63%)	0.4
Dilative cardiomyopathy	25/64 (39%)	11/22 (50%)	4/15 (27%)	10/27 (37%)	0.4
LV-EF (%)	30.8 ± 7.3	29.3 ± 6.9	30.0 ± 2.3	32.4 ± 8.1	0.2
NYHA I	0/64	0/22	0/15	0/27	
NYHA II	18/64 (28%)	5/22 (23%)	4/15 (23%)	9/27 (33%)	0.5
NYHA III	46/64 (72%)	17/22 (77%)	11/15 (73%)	18/27 (67%)	0.5
NYHA IV	0/64	0/22	0/15	0/27	
Primary prevention	31/64 (48%)	17/22 (77%)	0/15	14/27 (52%)	0.2
CRT-pacemaker	7/64 (11%)	1/22 (9%)	1/15 (7%)	5/27 (22%)	0.4
Medication					
Beta-blocker	64/64	22/22	15/15	27/27	
ACE-I/ARB	45/64 (70%)	14/22 (64%)	10/15 (67%)	21/27 (78%)	0.3
ARNI	19/64 (30%)	8/22 (36%)	5/15 (33%)	6/27 (22%)	0.7
MRA	50/64 (78%)	16/22 (73%)	11/15 (73%)	23/27 (85%)	0.3
Diuretics	53/64 (83%)	20/22 (90%)	11/15 (73%)	23/27 (85%)	0.7
Amiodarone	12/64 (19%)	0/22	8/15 (53%)	4/27 (15%)	0.1
Flecainide	0/64	0/22	0/15	0/27	
Oral anticoagulants	26/64 (41%)	8/22 (36%)	10/15 (67%)	8/27 (30%)	0.7
Antiplatelet therapy	31/64 (41%)	9/22 (41%)	5/15 (33%)	17/27 (63%)	0.2
Type of ventricular tachycardia					
Non sustained VT			6/15 (40%)		
VT below detection threshold			9/15 (60%)		

*ACE-I*, angiotensin-converting-enzyme-inhibitor; *ARB*, angiotensin II receptor blocker; *ARNI*, angiotensin II receptor blocker neprilysin inhibitor; *BMI*, body mass index; *LV-EF*, left ventricular ejection fraction; *MRA*, mineralocorticoid-receptor antagonist; *NYHA*, New York Heart Association functional classification of heart failure; *MRA*, mineralocorticoid-receptor antagonist; *VT*, ventricular tachycardia

### Procedural characteristics and outcome

In 22 patients, 26 ectopic foci were successfully ablated. In the majority of patients (18, 82%), a singular focus was treated, and in the remaining 18%, $\ge$ 2 foci were targeted. The mean procedure duration for PVC ablation was 146.5 ± 44.8 min. with a mean fluoroscopy time of 16.9 ± 9.1 min. The local activation at the successful ablation site was − 32.8 ± 11.9 ms. Pace mapping achieved a mean template matching percentage of 94 ± 4.5%. The majority of ectopic foci were ablated in the left ventricle (LV) and aorta with the right ventricle (RV) only being the source of the PVC in 3 patients (12%). The most frequent PVC locations were aorta (32%) and aortomitral continuity (19%). The detailed distribution of all sites of origin is provided in Table 2.

**Table 2:** Sites of successful ablation of ventricular ectopy

Site of origin	*n* (%)
Left ventricle	
LV — outflow tract	4/26 (15%)
Aortomitral continuity	5/26 (19%)
LV — summit	4/26 (15%)
LV — septum	3/26 (12%)
Posterior papillary muscle	1/26 (4%)
Aorta	
Left coronary cusp	4/26 (15%)
Right coronary cusp	1/26 (4%)
Non-coronary cusp	1/26 (4%)
Right ventricle	
RV — outflow tract	3/26 (12%)

*LV*, left ventricle; *RV*, right ventricle

Acute success was accomplished in 20/22 patients (91%). Of those, in 13/22 patients (59%), freedom from any PVCs was achieved, and in 7/22 patients (32%), the focus of the clinical ectopy was ablated. In 2/22 patients with PVC originating in the LV summit, no permanent PVC suppression was achieved. During a subsequent mean follow-up period of 7.7 ± 6.5 months, all CRT patients underwent 2.3 ± 0.9 consecutive follow-up visits evaluating the impact on BiVP.

All 15 patients undergoing VT ablation were inducible for VT at time of the procedure. In all patients, VT originated in the LV and the majority of patients were inducible for a single VT (60%). None of the induced VTs was hemodynamically tolerated during sedation. Consequently, all VTs were ablated following a substrate-based approach. The mean procedure duration was 196.6 ± 48.7 min and the fluoroscopy time was 26.6 ± 9.5 min. Acute success was accomplished in 14 (93%) patients undergoing VT ablation. Of those, in 12/15 (80%), abolishment of all VTs could be achieved and 2/15 (13%) were no longer inducible for the clinical VT.

### Complications

After PVC ablation, two access site complications occurred, one requiring transfusion. Both resolved with manual compression. One patient developed a pericardial effusion that resolved spontaneously.

Following VT ablation, one pericardial effusion requiring drainage occurred after extensive ablation. No procedure-related death or permanent injury occurred.

### Intensified medical therapy

In 23/27 patients, a preexisting betablocker therapy was intensified aiming for PVC suppression and optimization of BiVP. Of those, in 15/23 patients (65%), the recommended maximum daily dosage was reached. In another 8/23 patients (35%), the target dosage could not be obtained, due to hemodynamic intolerance or non-compliance. A small cohort (4/27) of patients received anti-arrhythmic drug treatment with amiodarone. No therapy-related complications were observed.

### Effect of different PVC treatment on biventricular pacing, LV-EF and NYHA class

The overall baseline BiVP was 90.5 ± 4.6% impaired by frequent ventricular ectopy. For BiVP optimization, 27/49 patients (55%) received intensified medical treatment and 22/49 patients (45%) underwent catheter ablation of ventricular ectopy. Overall, an increase of BiVP percentage points of 8.8% (range − 5 to + 47.6%) at 6-month follow-up was achieved, resulting in a mean BiVP of 96.5 ± 2.7%. In 23 patients (47%), the target range of a BiVP ≥ 98% was reached and even ≥ 99% was achieved in 6 patients (12%). The functional status of 16/49 patients (33%) improved from NYHA class III to NYHA class II (Table 3).

**Table 3:** Impact of clinical routine therapy of ventricular arrhythmia on biventricular pacing, functional status and LV-EF. Means and standard deviation or absolute numbers and percentage are shown. CRT, Cardiac resynchronization therapy. BiVP, biventricular pacing. LV-EF, left ventricular ejection fraction. NYHA, New York Heart Association functional classification of heart failure

Parameter	Overall	VT ablation group	PVC ablation group	Intensified medical therapy group	*p*-value
BiVP (%) pre-CRT optimization	88.1 ± 10.9	80.9 ± 14.8	88.1 ± 5.0	92.4 ± 3.2	0.001
BiVP (%) post-CRT optimization	96.7 ± 2.6	97.4 ± 3.3	98.2 ± 1.2	95.5 ± 2.9	0.001
Increase in biventricular pacing (%)	8.8 (range -5.0 to 47.6)	16.3 (range 3.3 to 32.7)	9.9 (range 1.2 to 47.6)	3.2 (range -5.0 to 10.7)	< 0.001
Biventricular pacing ≥ 98%	30/64 (47%)	9/15 (60%)	16/22 (73%)	6/27 (22%)	0.001
Biventricular pacing ≥ 99%	12/64 (19%)	6/15 (40%)	5/22 (23%)	1/27 (4%)	0.07
LV- EF (%) pre-CRT optimization	30.8 ± 7.3	29.3 ± 6.9	30.0 ± 2.3	32.4 ± 8.1	0.2
LV-EF (%) post-CRT optimization	31.3 ± 6.8	29.5 ± 6.6	31.5 ± 1.3	32.8 ± 7.9	0.1
NYHA class pre-CRT-optimization					
NYHA I	0/64	0/15	0/22	0/27	
NYHA II	18/64 (28%)	4/15	5/22 (23%)	9/27 (33%)	0.5
NYHA III	46/64 (72%)	11/15	17/22 (77%)	18/27 (67%)	0.5
NYHA IV	0/64	0/15	0/22	0/27	
NYHA class Post-CRT-optimization					
NYHA I	0/64	0/15	0/22	0/27	
NYHA II	42/64 (66%)	12/15(80%)	17/22 (77%)	11/27 (41%)	0.01
NYHA III	22/64 (34%)	3/15 (20%)	5/22 (23%)	16/27 (59%)	0.01
NYHA IV	0/64	0/15	0/22	0/27	
Improvement in NYHA class	24/64 (38%)	8/15 (53%)	12/22 (55%)	2/27(7%)	0.003
Follow-up	7.7 ± 6.5	9.2 ± 8.4	9.6 ± 7.0	5.2 ± 3.9	0.01

**Fig. 2:**
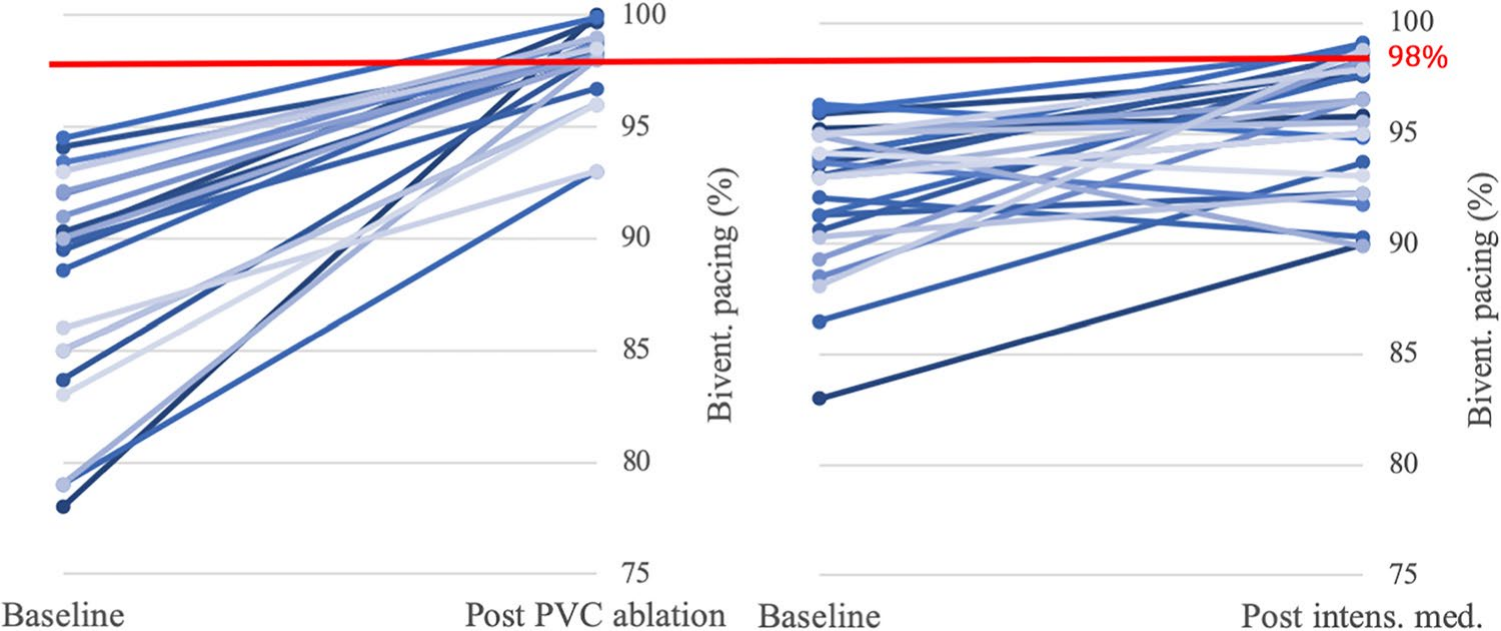
Impact of PVC ablation and intensified medical treatment biventricular pacing percentage. After PVC ablation an overall increase of 9.9 ± 4.8% was achieved resulting in a mean BiVP of 98.2 ± 1.2%. In 16/22 patients (73%), the target range of a BiVP percentage ≥ 98% was reached. After an intensified treatment, an increase of 3.2% (range − 5 to + 10.7%) was achieved resulting in a mean BiVP of 95.5 ± 2.9%. In 6 pts (22%), the target range of a BiVP percentage ≥ 98% was reached

When comparing the therapeutic interventions (Table 3), the baseline BiVP was lower (88.1 ± 5.1% vs. 92.4 ± 3.2%; *p* = 0.001) in the catheter ablation group. Intensified medical therapy alone increased the BiVP by 3.2% (range − 5.0 to 10.7%) while catheter ablation of PVC was significantly more effective (*p* =  < 0.001) to improve BiVP resulting in a mean gain of BiVP percentage of 9.9% (range 1.2 to 47.6%). Furthermore, in 5/27 patients (19%) in the medical treatment group, the BiVP percentage decreased further by − 2.2 ± 1.6% (Fig. 2).

Although the baseline BiVP was higher in the medical treatment group, more patients in the catheter ablation group reached the range of optimal BiVP (Fig. 2). A BiVP ≥ 98% was achieved in 16/22 patients (73%) after PVC ablation compared to 6/27 patients (22%) after intensified medical treatment (*p* =  < 0.001).

Irrespective of the treatment group, no significant changes were seen in left ventricular function at 6-month follow-up: LV-EF was 30.0% ± 2.3% pre-ablation vs. 31.5% ± 1.3% post-ablation (*p* = 0.3) in the PVC ablation group and 32.4 ± 8.1 pre-medication vs. 32.8% ± 7.9% post-medication (*p* = 0.8) in the medical therapy group.

**Fig. 3:**
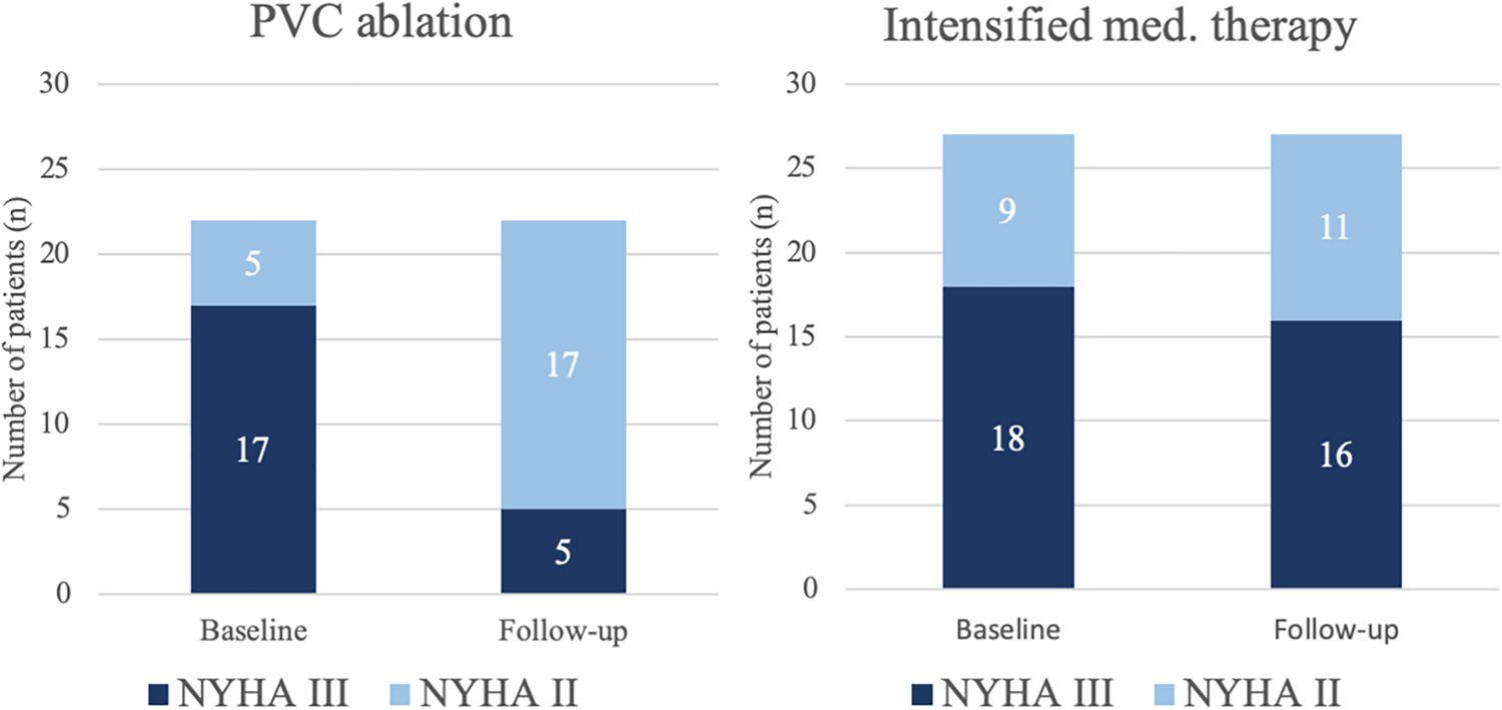
Impact of PVC ablation and intensified medical therapy on NYHA class. After PVC ablation, the functional status of 12/22 patients (55%) improved from NYHA class III to NYHA class II. After an intensified medical treatment, the functional status of 2/27 patients (7%) improved from NYHA class III to NYHA class II

Regarding the impact on heart failure symptoms, patients of the PVC ablation group had better improvement in functional status. While after PVC ablation heart failure symptoms improved from NYHA class III to NYHA class II in 12/22 patients (55%), medical treatment led to an improvement in NYHA class in 2/27 patients (7%; *p* = 0.003; Table 3, Fig. 3).

### Effect of VT ablation on biventricular pacing, LV-EF and NYHA class

All patients with non-sustained VTs in the device Holter or sustained VT below detection threshold underwent catheter ablation. The baseline BiVP was 80.7 ± 15.1%. VT ablation led to an increase of BiVP of 16.3% (range 3.3 to 32.7%) at 6-month follow-up. Following VT ablation, a BiVP ≥ 98% could be achieved in 9/15 patients (60%) and ≥ 99% in 6/15 patients (Table 3). No changes in LV-EF were seen either at 6-month follow-up (29.3% ± 6.9% pre-ablation vs. 29.5% ± 6.6% post-ablation; *p* = 0.8) but after VT ablation and BiVP optimization, 8/15 patients (53%) stated an improvement in functional status and improved from NYHA class III heart failure symptoms to NYHA class II (Table 3).

## Discussion

This non-randomized evaluation of different treatment options of ventricular arrhythmia to improve cardiac resynchronisation therapy provides several important findings.

In general, measures taken in routine clinical practice to improve BiVP, whether interventional or pharmacological, are effective. They lead to an improvement in heart failure symptoms, even in patients with minor or moderate BiVP reduction.

Most importantly, ablation of ventricular ectopy is a safe and effective tool to optimize CRT in patients with reduced BiVP. The ablation of predominately complex left ventricular ectopy led to a pronounced increase in BiVP, due to effective PVC suppression. Despite the lower BiVP at baseline, after PVC ablation, more patients had CRT devices operating in the target zone of BiVP ≥ 98% and in more in patients, a subsequent improvement in NYHA class was observed. Whether ablation is truly more effective than medical treatment, or this observation was the result of a selection bias, remains unclear.

Furthermore, VT ablation resulted in an increase in BiVP accompanied by an improvement in functional status.

### The importance of biventricular pacing percentage

Cardiac resynchronisation therapy is essential for heart failure patients with wide QRS complex. It improves quality of life, cardiac function, and, most importantly, heart failure mortality and morbidity [2, 15]. Its success is strongly linked to BiVP. Reduced BiVP is a common finding of CRT follow-up [4]. Large real-world cohorts showed rates of 40.7% of CRT patients with a relevant reduction of BiVP [6]. Considering these high rates of reduced BiVP in the context of data by Ruwald et al., demonstrating that every 1% increase in BiVP is associated with a 6% risk reduction in heart failure death emphasizes the importance of BiVP optimization [4].

The loss of CRT pacing is often a result of intrinsic ventricular activation [6]. If this occurs due to inappropriate programming, it may easily be corrected. Arrhythmia-induced BiVP reduction on the other hand requires a more complex therapeutic approach [16]. Atrial fibrillation commonly leads to a drastic dip in BiVP, facilitating an easy diagnosis and swift therapeutic action [17].

In contrast, ventricular arrhythmia including ventricular ectopy leads to a less pronounced reduction with an average BiVP of 85 to 97% [6]. However, this small decrease in BiVP may lead to deferred therapeutic actions and negative implications for patients’ outcome.

### Impact of ventricular ectopy on CRT

Frequent ventricular ectopy can cause or worsen heart failure [8]. Catheter ablation of PVC in heart failure patients has been shown to improve functional status and cardiac output and most recently to reduce morbidity and mortality [8].

Besides its general impact on heart-failure, frequent ventricular ectopy in CRT patients is known to have a direct impact on therapy success. Frequent PVC is associated with non-response, lower probability of LVEF recovery, and worse outcome. This is mainly driven by a reduction of biventricular pacing [10, 18].

### Antiarrhythmic drug therapy for PVC suppression in CRT patients

There are two historic studies available evaluating the therapeutical effect of amiodarone [19] and sotalol [20] on PVC in patients with structural heart disease. Both, accompanied by relevant side effects, showed a positive effect for the respective substance. But regarding specific antiarrhythmic drug therapy for PVC suppression in CRT patients, there is, to the best of our knowledge, no prospective or randomized data available. There is only one observational study available by Akerström et al. reporting that neither intensification of beta blocker therapy nor initialization of an amiodarone therapy had a significant impact on the PVC burden in CRT patients [21]. Consequently, due to the unclear benefit and frequent side effects of amiodarone and sotalol, an intensification of a preexisting beta blocker therapy, as conducted in this study, is currently the first therapeutical measure taken in routine clinical practice for PVC suppression in CRT patients [21].

### Ablation of complex ventricular ectopy for CRT optimization

Data on interventional treatment strategies for CRT patients with ventricular arrhythmia and BiVP reduction is also sparse. In patients with non-response to CRT and a high PVC burden with a severe reduction in BiVP, Lakkireddy et al. showed that PVC ablation leads to a significant increase in BiVP along with improvement in cardiac function and CRT response [10]. But, when compared to real-world cohorts, the BiVP percentage in this study was substantially lower. Ventricular ectopy in CRT patients tends to cause a smaller decrease in BiVP to around 90% [6]. Therefore, in clinical routine practice, a relevant number of patients, irrespective of the initial response status, either present a certain pre-implantation PVC burden or develop ventricular ectopy over time. This PVC burden inevitably leads to a small or moderately reduced BiVP percentage, which nevertheless requires treatment 13,18. But to the best of our knowledge, there is no data available favoring either medical therapy or catheter ablation for PVC treatment in this group of patients.

The present study’s results indicate that medical and interventional measures taken to improve BiVP in this particular cohort are both effective. In this real-world cohort with moderately impaired BiVP, PVC ablation was safe and increased BiVP. It was attended by an improvement in functional status. The effects of medical therapy on BiVP were less pronounced. Randomized trials are needed to compare the efficacy of both approaches.

Consequently, as even the slightest improvement in BiVP improves the outcome of CRT patients, present data suggest that measures to improve BiVP, even at rates of light impairment, should be employed promptly and patients should be followed up closely [4].

## Limitations

Due to its retrospective character, present study should be considered explorative and hypothesis generating. The main shortcoming is that patients were treated by a medical or interventional approach at the physicians’ discretion. Due to the lack of randomization, firm conclusions in the comparison between the two treatment modalities are therefore not possible. Although there were no differences between both PVC treatment groups, there is no information available regarding the PVC morphology or number of PVC morphologies in the medical therapy group. A resulting treatment bias can therefore not be excluded.

Furthermore, only a small number of patients (18%) underwent ablation of multifocal PVC. Application of the present study results on CRT patients with multifocal PVC is therefore limited. Patients in the medical therapy group were predominately treated with intensification of a preexisting betablocker therapy. Consequently, the low use of amiodarone or sotalol may have contributed to the reduced efficacy of PVC suppression in the medical therapy group. A further comparison between catheter ablation and specific antiarrhythmic drug treatment is therefore needed in the future.

But considering the encouraging results, the lack of published data, and its potential impact on clinical routine practice, it is reasonable to assume that catheter ablation of ventricular arrhythmia may be a potent tool to increase biventricular pacing. A randomized study is needed to confirm these findings.

## Conclusion

In this retrospective, non-randomized analysis of CRT patients with reduced BiVP, both intensified medical therapy and catheter ablation of ventricular arrhythmia effectively increased BiVP. In the PVC ablation group, a higher increase in BiVP was observed and more patients improved in NYHA class. Thus, catheter ablation of ventricular arrhythmia appears to be an effective tool to optimize CRT. Given the impact on heart failure prognosis, it seems reasonable to consider catheter ablation in CRT patients with reduced BiVP early. These findings need to be confirmed prospectively.
